# Psychosocial Determinants of Insomnia in Adolescents: Roles of Mental Health, Behavioral Health, and Social Environment

**DOI:** 10.3389/fnins.2019.00848

**Published:** 2019-08-09

**Authors:** Yi-Ping Hsieh, Wei-Hsin Lu, Cheng-Fang Yen

**Affiliations:** ^1^Department of Social Work, University of North Dakota, College of Nursing and Professional Disciplines, Grand Forks, ND, United States; ^2^Department of Psychiatry, Ditmanson Medical Foundation, Chia-Yi Christian Hospital, Chiayi City, Taiwan; ^3^Department of Psychiatry, Kaohsiung Medical University Hospital and School of Medicine, Graduate Institute of Medicine, Kaohsiung Medical University, Kaohsiung, Taiwan

**Keywords:** insomnia, suicidality, alcohol abuse, family support, school connectedness, favorable neighborhood, mental health, adolescent

## Abstract

The theoretical explanation of human problems is derived from the complex interplay of psychological, social, economic, political, and physical factors.

**Aims:**

This study examined the roles of behavioral health (i.e., alcohol abuse and suicidality) and social environment (i.e., family support, school connectedness, and favorable neighborhood) and mental health [i.e., depression, anxiety, and attention deficit hyperactivity disorder (ADHD)] in predicting insomnia in adolescents in an ecological perspective.

**Methods:**

Approximately 6445 high school students in Taiwan were administered an anonymous self-report survey. Hierarchical multiple regression was performed to examine how multidimensional social environment, behavioral health, and mental health factors were associated with insomnia in adolescents.

**Results:**

The prevalence rate of insomnia in the sample was 30%. The results indicated that alcohol abuse (β = 0.04), suicidality (β = 0.06), depression (β = 0.29), anxiety (β = 0.14), and ADHD (β = 0.11) were positively associated with insomnia (*p* < 0.001), whereas family support (β = −0.06), school connectedness (β = −0.05), and favorable neighborhood *(*β = −0.10) were negatively associated with insomnia (*p* < 0.001). Sex did not predict insomnia, but age was positively associated with insomnia (β = 0.09, *p* < 0.001). Among all predictors of insomnia in the study, mental health factors, especially depression, play a major role on insomnia among adolescents, and is as much important as social environment factors.

**Conclusion:**

This study demonstrated how both psychosocial variables (social environment and behavioral health) and psychological symptoms were associated with insomnia in adolescents when the demographic variables (sex and age) were controlled and provided valuable information and evidence for clinicians, social workers, and health professionals who provide support to adolescents with insomnia. Applying an ecological approach in practice can aid in understanding at individual, family, school, and community levels and in identifying the strengths and weaknesses of their interactions with each other.

**Implications:**

This perspective enables practitioners in effectively treating problems and addressing the needs of the various levels, including the individual, family, school, and the broader community. Thus, prevention and intervention of insomnia in adolescents should focus on multidimensional risk and protective factors, including mental health, behavioral health, and social environment, in the context of an ecological system.

## Introduction

Insomnia is a subjective complaint, defined as difficulty in falling asleep, trouble in staying asleep, or having poor sleep quality (Ohayon, 2002). In Taiwan, >25% (*n* = 36,743) of adults and 18.7% adolescents (aged 12–18 years) experienced insomnia (Kao et al., 2008; Huang et al., 2010). The most common insomnia type was the difficulty in initiating sleep, followed by early morning awakening and difficulty in maintaining sleep (Kao et al., 2008). In the United States, the age-adjusted prevalence of insomnia was 18.8% (Cunningham et al., 2015) and was 19.3% among young children and preadolescents (Calhoun et al., 2014).

Why study insomnia in adolescents? Insufficient and unsatisfactory sleep in adolescents is associated with adverse outcomes across various areas of youth development, including physical health (e.g., inflammation), cognitive and behavioral function, school performance, delinquency, substance use, depression, diminished quality of life, and even suicide ideation and self-harm behavior (Roberts et al., 2001; Sadeh et al., 2003; Curcio et al., 2006; Clinkinbeard et al., 2011; Mueller et al., 2011; Wong et al., 2011; Pasch et al., 2012; Park et al., 2016; Wang et al., 2017). Moreover, sleep is critical for all areas of adolescent development. However, sleep disruption and insufficient sleep are frequent among youth (Calhoun et al., 2014). Therefore, in addition to previous studies focusing on physical conditions such as chronic painful physical condition as a major contributive factor for insomnia (Ohayon, 2005), it is important for the present study to identify psychological, social, behavioral, and contextual barriers and aids to induce good sleep.

Considering the person-in-environment perspective, it is important to understand an individual and individual behavior in light of the environment contexts in which that person lives and acts. Thus, in addition to mental health [i.e., depression, anxiety, and attention deficit hyperactivity disorder (ADHD)], the current study also examined the roles of behavioral health (i.e., alcohol abuse and suicidality) and social environment (i.e., family support, school connectedness, and favorable neighborhood) simultaneously in predicting insomnia in adolescents. First, regarding behavioral health, alcohol abuse and suicidality are potential barriers to good sleep and risk factors for insomnia. Concerning alcohol abuse, studies have found that chronic alcoholic exposure can cause sleep disruptions, which is manifested by increasing sleep-onset latency and wakefulness (Thakkar et al., 2015). In addition, alcohol often interacts with sleep deprivation and sleep restriction to exacerbate daytime sleepiness and alcohol-induced performance impairments (Roehrs and Roth, 2001). Regarding suicidality, a meta-analysis concluded that sleep disturbance was significantly associated with an increased relative risk of suicidal ideation, suicide attempt, and suicide (Pigeon et al., 2012). Similarly, a longitudinal study identified variability in sleep timing and insomnia as warning signs of suicide ideation (Bernert et al., 2017). However, little research examined whether higher levels of suicidality was associated with higher levels of sleep problems.

Second, regarding psychosocial and contextual factors in the ecological system (Bronfenbrenner, 1979), family support, school connectedness, and favorable neighborhood are potential aids to good sleep and preventive factors of insomnia. Concerning family support, having strained family relationships was associated with more troubled sleep, whereas supportive family relationships are associated with less disturbed sleep, particularly in individuals who are in frequent contact with the family members (e.g., adolescents) (Ailshire and Burgard, 2012). Sleep troubles are most evident when family relationships are highly strained and provide inadequate emotional support. In the context of family stress, Tsai et al. (2018) reported that parental support was associated with longer sleep duration, less sleep variability, and less time spent awake during the night. It suggested that cohesive family relationships and support provide a sense of stability and security that is necessary for healthy sleep. Regarding the school-level predictors of insomnia, most of the research focuses on the effects of insomnia on school performance or the link between academic stress and insomnia (Yan et al., 2018). However, not much research has explored the relationship between school connectedness and insomnia among adolescents. In a sample of Chinese male adolescents, although sleep problems at the beginning of the school year predicted lower levels of school connectedness at the end of the school year, but school connectedness at the beginning of the school year did not predict sleep problems at the end of the school year (Bao et al., 2018). In a sample of Latino and Asian adolescents, a sense of school belongingness buffered the negative effect of overt discrimination on sleep (Huynh and Gillen-O’Neel, 2016), which suggested that school belongingness represents a positive support for adolescents that induce good sleep. Regarding community-level predictors of insomnia, adverse neighborhood and social environment (e.g., high disorder, low safety and social cohesion, violence, and noise) were associated with a higher prevalence of short sleep and insomnia (DeSantis et al., 2013; Simonelli et al., 2017). Moreover, the results of a global analysis of six countries (*n* = 39,590) indicated that perceived neighborhood safety is negatively associated with insomnia symptoms and poor sleep quality in the past 30 days (Hill et al., 2016). Therefore, favorable neighborhood is a potential preventive factor of insomnia.

Third, regarding mental health, depression, anxiety, and ADHD are potential barriers to good sleep and risk factors for insomnia. Concerning depression, the relationship between insomnia and depression was found to be reciprocal (Roberts and Duong, 2013; Lovato and Gradisar, 2014). Individuals with insomnia have a twofold risk of depression compared with those with no sleep difficulties (Baglioni et al., 2011). By contrast, adolescents with depression experienced significantly more wakefulness in bed, lighter sleep, and more subjective sleep disturbance (Lovato and Gradisar, 2014). Depressive symptoms in adolescents significantly predicted sleep problem development and persistence at a 4-year follow-up (Patten et al., 2000). Adolescents who reported depressive symptoms were 50% more likely to develop sleep problems than those who did not report any symptoms. Regarding anxiety, a systematic review has reported a one-way relationship where anxiety predicted excessive daytime sleepiness (Alvaro et al., 2013). Another study in youth found that anxiety preceded insomnia 73% of the time after adjustments for gender, race, and ethnicity (Johnson et al., 2006). Similarly, an epidemiological study in India revealed that anxiety was independently associated with insomnia after adjustments for other variables (Khan et al., 2018). Regarding ADHD, a study in the Netherlands showed that 43% of patients with ADHD reported significant insomnia (odds ratio = 2.66) and 41% reported short sleep duration (Wynchank et al., 2018). Another study in Ankara, Istanbul found that probability of insomnia was higher (2.7-fold) among those with probable ADHD, and both inattentiveness and hyperactivity dimensions of ADHD was related to insomnia severity (Evren et al., 2019). A clinical review paper indicated high rates of parental reports of sleep disturbances in children with ADHD (Cohen-Zion and Ancoli-Israel, 2004). Notably, children with ADHD have a fivefold increase in sleep problems than children without ADHD.

Therefore, this study expands on the previous research by simultaneously examining multidimensional psychosocial determinants, such as behavioral health (i.e., alcohol abuse and suicidality), social environment (i.e., family support, school connectedness, and favorable neighborhood), and mental health (i.e., depression, anxiety, and ADHD) to determine their association with insomnia in adolescents in the context of an ecological system. Concordant with previous research, we hypothesized that alcohol abuse, suicidality, depression, anxiety, and ADHD would be the risk factors for insomnia in adolescents, after control for sex and age. By contrast, family support, school connectedness, and conducive neighborhood would be the preventive factors associated with insomnia.

## Materials and Methods

### Participants

The participants in this study were enrolled from the 2009 Project for the Health of Children and Adolescents in Southern Taiwan, a research program studying the mental health status of children and adolescents living in four counties and three metropolitan areas in southern Taiwan (Yen et al., 2010a). In 2009, there were 254,130 students in 205 junior high schools and 202,883 students in 143 senior high/vocational schools in this area. On the basis of the definitions of urban and rural districts in the Taiwan-Fukien Demographic Fact Book (Ministry of the Interior, 2002), a stratified random sampling strategy was used to ensure that there was proportional representation of districts and schools. Two-stage random cluster sampling was used to select schools and classes for the study. Five junior high schools and five senior high or vocational schools were randomly selected from urban districts. Similarly, five junior high schools and four senior high or vocational schools were randomly selected from the rural districts. The classes were randomly selected from these schools. All students in the selected classes were invited to participate in the study, except the students with special needs. Subsequently, 6703 students from grades 7 to 12 participated in the study.

### Procedure

This study was approved by the Institutional Review Board (IRB) of Kaohsiung Medical University before sampling and data collection. The research presents no more than minimal risk of harm to subjects and involves no procedures for which written consent is normally required outside of the research context. And, the data were recorded in a manner that individuals cannot be identified in the anonymous survey. The waiver of written parental consent form did not adversely affect the rights and welfare of the subjects and was approved by IRB. The passive parental consent was used. Prior to the administration of the survey, parents/guardians of all student participants received written notice letter that their child would be recruited to participate in the study and explaining the study purpose and procedure, emphasizing the voluntary and confidential nature of the study. Parents were given the opportunity to exclude their daughter/son from participation in the study and asked to respond within 3 days. Students took the letter home for their parents or guardians, who were given a choice to call the researchers, write in the school communications book, or ask their children to directly, all convey to their refusal for their children to join the study. In addition, the students were given the liberty to refuse participation in this study by returning blank questionnaires along with those from other students.

### Measures

Based on the ecological system theory, we included three layers of social environment as predictors in the study: family support, school connectedness, and favorable neighborhood. We assessed the students’ experiences with insomnia, mental health (i.e., depression, anxiety, and ADHD), behavioral health (i.e., alcohol use and suicidality), and social environment (i.e., family support, school connectedness, and favorable neighborhood) by using the self-reported surveys. A pilot study (*n* = 76) was conducted to ensure adequate reliability of all research instruments by performing a 2-week test–retest reliability.

#### Insomnia

The 8-item Athens Insomnia Scale (AIS-8) was used to assess the severity of subjective insomnia over the preceding month (Soldatos et al., 2000). The items of the AIS-8 correspond to the taxonomies for the diagnosis of insomnia per the International Classification of Diseases, Tenth Edition. The first five items assess difficulty with sleep induction, awakening during the night, early morning awakening, total sleep time, and overall quality of sleep. The last three items assess the next-day consequences of any insomnia, including problems with a sense of well-being, functioning, and daytime sleepiness. The Taiwanese version of the AIS-8 scale has been validated in previous study (Chung et al., 2011; Sun et al., 2011). Each item of the AIS-8 was rated on a four-point rating scale from 0 to 3 with a higher score indicating severe insomnia. The mean score was calculated. The Cronbach’s alpha in the present study was 0.69, and the 2-week test–retest reliability was 0.72 (*p* < 0.001).

#### Depression

The 20-item Mandarin-Chinese version of the Center for Epidemiological Studies-Depression (CES-D) Scale was used to assess the frequency of depressive symptoms in the previous week (Radloff, 1977; Chien and Cheng, 1985). Each item of the CES-D was rated on a 4-point rating scale from 0 to 3. A higher score indicated severe depression. The mean score was calculated. The Cronbach’s alpha for the CES-D was 0.89, and the 2-week test–retest reliability was 0.78.

#### Anxiety

We used the Taiwanese Version of the Multidimensional Anxiety Scale for Children (MASC-T) to assess the participants’ self-reported anxiety symptoms (March, 1997; Yen et al., 2010b). The MASC-T consisted of 39 items answered on a 4-point Likert scale from 0 to 3. A higher score represented a severe level of anxiety. The mean score was calculated. The Cronbach’s alpha for the MASC-T in the present study was 0.89.

#### ADHD

An 18-item self-report ADHD scale was modified from the Vanderbilt ADHD Diagnostic Parent Rating Scale (Wolraich et al., 2003) and represented the 18 diagnostic symptoms of ADHD from the DSM-IV, Text Revision (American Psychiatric and Association, 2000). Items 1–9 measure inattention symptoms, and items 10–18 measure hyperactivity and impulsivity symptoms. Taiwanese version of ADHD scale has been validated in previous study (Gau et al., 2008). Participants rated their symptoms on a 4-point Likert scale from 0 to 3 with a higher score indicating symptom severity. The mean score was calculated. The Cronbach’s alpha was 0.88.

#### Alcohol Abuse

The 5-item alcohol abuse scale was adapted from the CRAFFT substance abuse screening test to assess problematic alcohol use in adolescents (Knight et al., 2002). We removed one item “CAR” to increase the reliability. The “yes” answer was scored as 1 and “no” as 0. A higher total score on the scale represented a severe level of alcohol abuse. The Cronbach’s alpha for the alcohol abuse scale was 0.65.

#### Suicidality

The 5-item questionnaire from the epidemiological version of the Kiddie Schedule for Affective Disorders and Schizophrenia (K-SADS-E; Puig-Antich and Chambers, 1978) was used to assess suicidal ideation and attempt in the preceding year. Questions were as follows: (1) “Has there ever been a period of 2 weeks or longer when you thought a lot about death, including thoughts of your death, somebody else’s death, or death in general?” (2) “Has there ever been a period of 2 weeks or longer when you had a desire to die?” (3) “Have you ever thought of attempting suicide?” (4) “Have you had a suicidal plan?” (5) “Have you ever attempted suicide?” Each question elicited a “yes” or “no” answer. In a previous study in Taiwan, the Cohen’s kappa coefficient of agreement (j) between participants’ self-reported suicide attempts and their parents’ reports was 0.54 (*p* < 0.001) (Tang et al., 2009). The Cronbach’s alpha in this study was 0.79.

#### Family Support

The 5-item Chinese version of the Family APGAR Index (Smilkstein, 1978; Chen et al., 1980) was applied to measure participants’ satisfaction with aspects of family support on a 4-point rating scale from 0 to 3. A higher score represented a higher level of family support. The mean score was calculated. The Cronbach’s alpha in the present study was 0.85.

#### School Connectedness

The level of connectedness to school was assessed based on five statements: “I enjoy my school life,” “I get along well with my teachers,” “I get along well with my schoolmates,” “I feel that I am popular,” and “I feel no interest to interact with friends or schoolmates (revise coded).” The participants scored the items on a 4-point Likert scale from 0 to 3, with a higher score indicating more agreement with the statements. To make the interpretation easier, we reverse-coded these items and calculated the sum score. After recoding, a higher score indicating a higher level of school connectedness. The mean score was calculated. The Cronbach’s alpha was 0.68.

#### Favorable Neighborhood

The 8-item favorable living environment scale was adapted from the Taiwanese Quality of Life Questionnaire for Adolescents (Fuh et al., 2005) to assess experiences in the preceding 2 weeks. A sample question was as follows: “Do you feel safe and protected in your home?” The participants reported their experiences on a 5-point rating scale from 0 to 4. A higher score indicated better quality of life over the preceding 2 weeks. The mean score was calculated. The Cronbach’s alpha in this study was 0.89.

### Analysis Plan

Statistical analysis was performed using the SPSS Statistical Package for Windows, version 24. First, descriptive statistics were used to assess the distribution of adolescents’ insomnia, behavioral health (i.e., alcohol abuse and suicidality), social environment (i.e., family support, school connectedness, and favorable neighborhood), and mental health (i.e., depression, anxiety, and ADHD) based on the ecological system theory. Second, we conducted correlational analyses using the Pearson coefficient of correlation to illustrate interrelationships between each of the variables. Third, we performed hierarchical regression to examine the effects of behavioral health, social environment, and mental health on insomnia in adolescents, after control for age and sex.

## Results

### Descriptive Statistics and Correlations

Overall, 6445 participants completed the research questionnaires without withdrawal, of which 52% were girls and 48% were boys. Table 1 presents the correlation coefficients of all study variables and the descriptive statistics of the means and standard deviations of the variables. The prevalence rate of insomnia in the sample was 30%, based on the optimal cut-offs for AIS-8 (Chung et al., 2011).

**TABLE 1:** Bivariate correlations, means, and standard deviations for variables in the models.

	**1**	**2**	**3**	**4**	**5**	**6**	**7**	**8**	**9**	**10**	**11**
1. Insomnia	–										
2. Sex	−0.05^∗∗^	–									
3. Age	0.13^∗∗^	−0.5^∗∗^	–								
4. Alcohol abuse	0.15^∗∗^	0.05^∗∗^	0.06^∗∗^	–							
5. Suicidality	0.33^∗∗^	−0.09^∗∗^	0.01	0.21^∗∗^	–						
6. Family support	−0.28^∗∗^	−0.10^∗∗^	−0.06^∗∗^	−0.15^∗∗^	−0.25^∗∗^	–					
7. SC	−0.32^∗∗^	−0.03^∗^	0.05^∗∗^	−0.08^∗∗^	−0.21^∗∗^	0.28^∗∗^	–				
8. FN	−0.36^∗∗^	0.08^∗∗^	−0.11^∗∗^	−0.11^∗∗^	−0.25^∗∗^	0.45^∗∗^	0.34^∗∗^	–			
9. Depression	0.55^∗∗^	−0.11^∗∗^	0.08^∗∗^	0.17^∗∗^	0.48^∗∗^	−0.37^∗∗^	−0.50^∗∗^	−0.43^∗∗^	–		
10. Anxiety	0.37^∗∗^	−0.21^∗∗^	−0.03^∗^	−0.01	0.26^∗∗^	0.02	−0.24^∗∗^	−0.19^∗∗^	0.52^∗∗^	–	
11. ADHD	0.38^∗∗^	0.06^∗∗^	0.09^∗∗^	0.15^∗∗^	0.24^∗∗^	−0.24^∗∗^	−0.26^∗∗^	−0.27^∗∗^	0.47^∗∗^	0.32^∗∗^	–
Mean	0.78	–	14.78	0.35	0.71	1.81	2.09	2.50	0.79	0.99	0.91
*SD*	0.39	–	1.81	0.81	1.27	0.70	0.47	0.74	0.49	0.40	0.44

*^∗^*p* < 0.05. ^∗∗^*p* < 0.01. SC, school connectedness; FN, favorable neighborhood; SD, standard deviation. Codes for sex are 1 = male, 0 = female.*

### Effects of Psychosocial Determinants on Insomnia

Prior to conducing a hierarchical multiple regression, the relevant assumptions of this statistical analysis were tested. Firstly, a sample size of 6445 was deemed adequate given 10 independent variables to be included in the analysis (Tabachnick et al., 2007). The assumption of singularity was also met as the independent variables were not a combination of other independent variables. An examination of correlations (Table 1) revealed that no independent variables were highly correlated, with the exception of depression and anxiety (*r* = 0.52). As the collinearity statistics (i.e., Tolerance and VIF) were all within accepted limits, the result indicated that multicollinearity was not a concern (Hair et al., 1998). The residual plots, the normal probability plot, and scatter plots indicated that the assumptions of normality, linearity, and homoscedasticity were all satisfied (Hair et al., 1998; Pallant, 2013; Supplementary Appendix 1). And, the value of Durbin–Watson test statistic was 2 that indicated the residuals were uncorrelated (Durbin and Watson, 1951). Hierarchical regression analyses were used to examine whether adolescents’ two behavioral health and three social environment factors, and three mental health factors were significant contributors to insomnia, after control for sex and age. Table 2 summarizes the results from the hierarchical regression. The demographic variables (sex and age) were entered into the regression model of insomnia in the first step (Model 1). Behavioral health and social environment variables (i.e., alcohol abuse, suicidality, family support, school connectedness, and favorable neighborhood) were entered into the regression model in the second step (Model 2). Mental health variables (i.e., depression, anxiety, and ADHD) were entered in the third and final step (Model 3).

**TABLE 2:** Summary of hierarchical regression analysis for variables predicting insomnia (*n* = 6,445).

	**Model 1**			**Model 2**			**Model 3**			**Model 3**
				
**Predictor variables**	***B***	***SE***	**β**	***B***	***SE***	**β**	***B***	***SE***	**β**	**95% CI**
**Step 1: Demographic**										
Child sex	−0.03	0.01	−0.04^∗∗^	−0.02	0.01	−0.02^∗^	0.01	0.01	0.02	−0.004–0.028
Child age	0.03	0.01	0.13^∗∗∗^	0.02	0.01	0.11^∗∗∗^	0.02	0.01	0.09^∗∗∗^	0.015–0.023
**Step 2: Behavioral health and social environment**										
Alcohol abuse				0.02	0.01	0.05^∗∗∗^	0.02	0.01	0.04^∗∗^	0.007–0.027
Suicidality				0.06	0.01	0.21^∗∗∗^	0.02	0.01	0.06^∗∗∗^	0.012–0.026
Family support				−0.05	0.01	−0.09^∗∗∗^	−0.04	0.01	−0.06^∗∗∗^	−0.048 to −0.022
School connectedness				−0.16	0.01	−0.20^∗∗∗^	−0.04	0.01	−0.05^∗∗∗^	−0.060 to −0.022
Favorable neighborhood				−0.10	0.01	−0.18^∗∗∗^	−0.05	0.01	−0.10^∗∗∗^	−0.065 to −0.040
**Step 3: Mental health**										
Depression							0.23	0.01	0.29^∗∗∗^	0.204–0.253
Anxiety							0.14	0.01	0.14^∗∗∗^	0.115–0.162
ADHD							0.10	0.01	0.11^∗∗∗^	0.080–0.121
**Fit test**										
Adjusted *R*^2^			0.02			0.24			0.36	
*R*^2^ change			0.02			0.22			0.12	

*Codes for sex are 1 = male, 0 = female. ^∗^*p* < 0.05, ^∗∗^*p* < 0.01, ^∗∗∗^*p* < 0.001.*

The results showed that adolescents’ sex and age significantly contributed to the regression model, *F*(2,6388) = 61.18, *p* < 0.001 and accounted for 2% of the variance in insomnia. Introducing two behavioral health and three social environmental variables significantly explained an additional 22% of the variance in insomnia and this change in *R*^2^ was significant, *F*(5,6383) = 371.98, *p* < 0.001. Finally, the addition of three mental health variables to the regression model explained an additional 12% of the variance in insomnia and this change in *R*^2^ was also significant, *F*(3,6380) = 403.69, *p* < 0.001 (Δ*R*^2^ = 0.12, *p* < 0.001). Taken together, in the final model, these demographic, behavioral health variables, social environment variables, and mental health variables significantly explained 36% of the variance in insomnia. The sum of square was 345.71 and the sum of square error was 610.86. A Mallow’s *Cp* value (*Cp* = 11) that was close to the number of predictors plus the constant indicated that the model produced relatively precise and unbiased estimates. The regression coefficients indicated that alcohol abuse (β = 0.04), suicidality (β = 0.06), depression (β = 0.29), anxiety (β = 0.14), and ADHD (β = 0.11) were positively associated with insomnia (*p* < 0.001), whereas family support (β = −0.06), school connectedness (β = −0.05), and favorable neighborhood *(*β = −0.10) were negatively associated with insomnia (*p* < 0.001). Therefore, adolescents who reported higher levels of alcohol abuse, suicidality, depression, anxiety, and ADHD were more likely to have insomnia. In contrast, adolescents who perceived higher levels of family support, school connectedness, and favorable neighborhood were less likely to have insomnia. Sex did not predict insomnia. Age was positively associated with insomnia (β = 0.09, *p* < 0.001). Older adolescents reported more insomnia than younger adolescents. Among all predictors of insomnia in the study, mental health factors, especially depression, play a major role on insomnia among adolescents, and is as much important as social environment factors. When applying stepwise elimination approach, depression was the best predictor that accounted for the most of the variance in insomnia [Δ*R*^2^ = 0.30, *p* < 0.001; *F*(1,6389) = 2775.41, *p* < 0.001], followed by ADHD and favorable neighborhood, and school connectedness accounted for the least of the variance in insomnia.

## Discussion

The present study extends the previous research regarding the impact of behavioral and mental health and social environment on insomnia in adolescents. This study addressed the increasing problems of insomnia in adolescents and added to the fund of knowledge by analyzing how multidimensional factors contribute to insomnia in adolescents at individual, family, school, and community levels in the ecological system. The results supported our hypotheses. First, we found empirical support that adolescent alcohol abuse and suicidality were positively associated with insomnia, after control for age, sex, and other factors. Second, we revealed that family support, school connectedness, and favorable neighborhood were negatively associated with adolescent insomnia, after control for demographics and other covariates. Third, we found that mental health problems such as depression, anxiety, and ADHD were positively associated with insomnia when controlling the demographics and other covariates. Both the behavioral health and social environment variables and the mental health variables explained the significant variance in the model.

### Behavioral and Mental Health and Insomnia

Regarding behavioral health problems, the study confirmed the relationship between alcohol abuse and insomnia among adolescents in Taiwan – concordant with previous research on adult samples (Chaudhary et al., 2015). Adolescents with alcohol abuse issues are at a higher risk of a chronic predicament of insomnia. The correlation between alcohol abuse and insomnia was also found in a Polish study sample that indicated >60% of alcohol-dependent patients screened positive for insomnia (Zhabenko et al., 2012), and insomnia was also found to be prevalent in 75% of alcohol-dependent adults in a US sample (Chaudhary et al., 2015). On the other hand, sleep disturbances may place adolescents without alcohol abuse issues at a high risk of alcohol abuse (Hasler et al., 2014). Furthermore, concordant with a previous research of an extensive school-based survey and a longitudinal study in the United States (Roberts et al., 2001; Wong et al., 2011), this study on a Taiwanese sample confirmed that suicidality was associated with insomnia among the adolescents, after accounting for depression, anxiety, PTSD, and alcohol abuse. Regarding mental health problems, most studies found strong relationships with insomnia in adults or older adults (Cho et al., 2008). For example, a Swedish study of a general population sample found that depression at its baseline is related to subsequent symptoms of insomnia (Jansson-Fröjmark and Lindblom, 2008). In addition, a prospective study in the United Kingdom on an adult sample revealed that depression predicted the incidence of insomnia at 1-year follow-up (Morphy et al., 2007). This study conducted in Taiwan showed that depression, anxiety, and ADHD significantly affected insomnia in adolescents, after control for the effects of the social environment and behavioral health.

### Social Environment and Insomnia

Considering that behavioral and mental health problems were the risk factors for insomnia, we examined the potential preventive factors in the three social environment levels that were related to low insomnia levels. This relationship has not been explored much previously. We hypothesized three preventive factors in the context of family, school, and community to represent the social environment in the ecological system. The results supported the hypotheses that higher family support, school connectedness, and a conducive neighborhood contributed to low risk of insomnia among adolescents. Based on the stress-buffering model, social support protected individuals from the negative psychological consequences of a wide range of stressful events that can help reduce insomnia (Cohen and Wills, 1985; Cohen et al., 2000). Family support and school connectedness are two essential types of social support for adolescents. Adolescents perceived a high level of social support when they felt that they were cared for, loved, held in high esteem, and were members of a network of mutual obligations. These perceptions enabled the adolescents to cope and adapt in life (Cobb, 1976), and consequently reduced sleep problems. Concordant with a previous US study on an adult sample (Ailshire and Burgard, 2012), we confirmed that perceived family support was associated with less insomnia in adolescents. The relationship between school connectedness and insomnia among the adolescents has not been researched much, but previous research suggested that school belongingness/connectedness may be a positive support for the adolescents (Huynh and Gillen-O’Neel, 2016). Our results support the hypothesis that adolescents who perceived higher school connectedness had fewer sleep problems, such as insomnia. Finally, our study on Taiwanese adolescents found that favorable neighborhood was associated with less insomnia, which was concordant with a previous research in the United States on adult samples that found higher prevalence of insomnia related to adverse neighborhood (Hill et al., 2009; DeSantis et al., 2013; Simonelli et al., 2017).

### Strengths and Limitations

A strength of our study is the use of an ecological perspective to examine the roles of mental health and multidimensional psychosocial determinants, such as behavioral health (i.e., alcohol abuse and suicidality) and social environment (i.e., family support, school connectedness, and favorable neighborhood), in insomnia in adolescents. Holistic thinking can provide a paradigm to understand how various systems and their interactions can contribute to an individual’s problem and behavior. Another strength is the large size of the study sample. By contrast, a limitation of the study is its cross-sectional design that can only draw conclusions regarding the correlation between the study variables and not interpret the cause-and-effect. Moreover, some of the variables may have a reciprocal relationship with insomnia. For example, depression predicts insomnia, whereas insomnia also predicts depression. Future research may be warranted to examine the reciprocal relationships between the psychosocial variables and insomnia and identify patterns of a negative cycle.

### Implications

The study provides valuable information and evidence for clinicians, social workers, and health professionals providing support to adolescents with insomnia. Applying an ecological approach in practice aids in understanding the issues at individual, family, school, and community levels and identify the strengths and weaknesses of the interactions between them. This perspective allows the practitioner to effectively treat problems and address the needs of the various levels, including the individual, family, school, and the broader community. The prevention and intervention of insomnia in the adolescents should focus on multidimensional risks and preventive factors, including mental health, behavioral health, and social environment in the context of an ecological system.

## Data Availability

The raw data supporting the conclusions of this manuscript will be made available by the authors, without undue reservation, to any qualified researcher.

## Ethics Statement

This study was approved by the IRB of Kaohsiung Medical University before sampling and data collection. Before conducting the study, we prepared a leaflet explaining the study purpose and procedure, emphasizing the voluntary and confidential nature of the study. Students took the leaflets home for their parents or guardians, who were given a choice to call the researchers, write in the school communications book, or ask their children to directly, all convey to their refusal for their children to join the study. In addition, the students were given the liberty to refuse participation in this study by returning blank questionnaires along with those from other students.

## Author Contributions

Y-PH: conceptualization, methodology, software, validation, formal analysis, investigation, resources, data curation, writing – original draft preparation, review and editing, and visualization. W-HL and C-FY: conceptualization, validation, investigation, resources, data curation, review and editing, supervision, project administration, and funding acquisition.

## Conflict of Interest Statement

The authors declare that the research was conducted in the absence of any commercial or financial relationships that could be construed as a potential conflict of interest.
