# Supplementary figures and images for: Psychosocial Determinants of Insomnia in Adolescents: Roles of Mental Health, Behavioral Health, and Social Environment
Source: Front Neurosci. 2019 Aug 9;13:848. doi: 10.3389/fnins.2019.00848 (PMC6696979; doi:10.3389/fnins.2019.00848)

Appendix 1


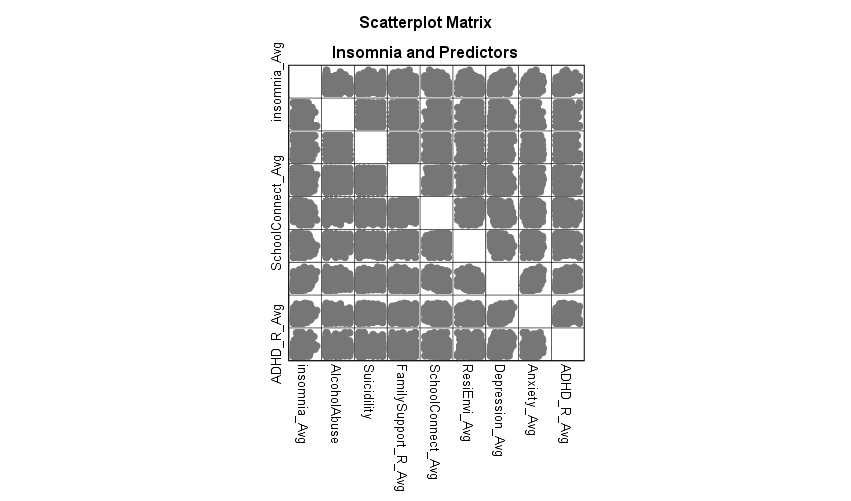


Residual Plots for Model 3:


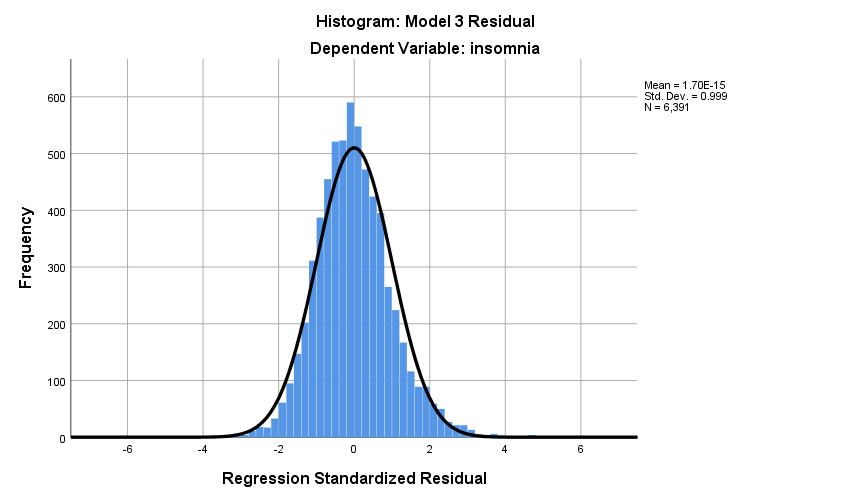


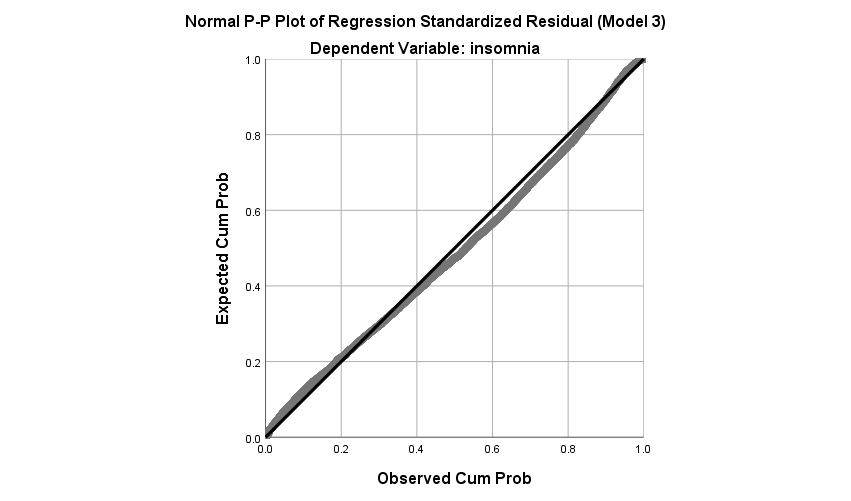


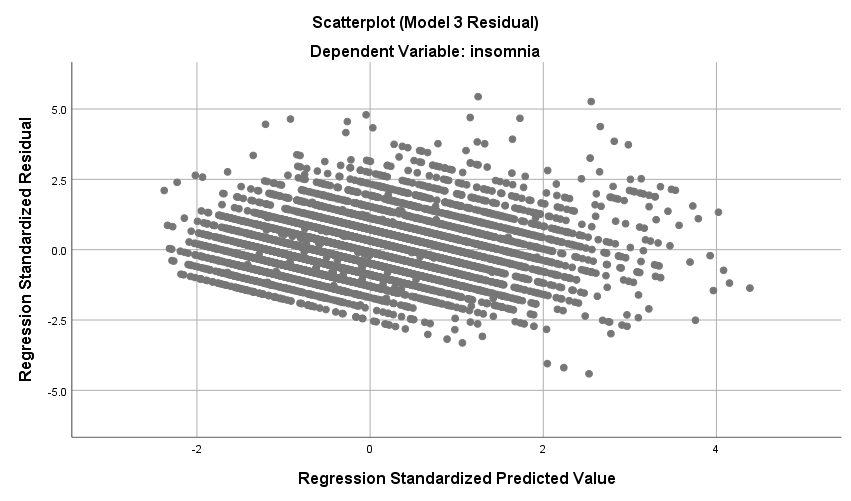

Supplement: Supplementary file 1 [file Table_1.DOCX]
